# Evolution of Whole-Cell Biosensor Detection Technology for PAHs and Their Halogenated Derivatives Driven by Performance Requirements

**DOI:** 10.3390/bios16070378

**Published:** 2026-07-10

**Authors:** Jingfang Zhang, Wenhui Mao, Shiqi Xia, Liangshu Hu, Mingzhang Guo, Huilin Liu

**Affiliations:** 1 Key Laboratory of Digital-Intelligence and Dynamic Perception for Food Quality of China Light Industry, Beijing Technology and Business University, Beijing 100048, China; 2431031036@st.btbu.edu.cn (J.Z.); 2431031095@st.btbu.edu.cn (W.M.); xiashiqi@st.btbu.edu.cn (S.X.); 2350201004@st.btbu.edu.cn (L.H.); 2 Beijing Laboratory for System Engineering of Carbon Neutrality, Beijing Municipal Education Commission, Beijing 100048, China; 3 BTBU-CAU Joint Laboratory of Synthetic Biology for Food, Nutrition and Health, Beijing Technology and Business University, Beijing 100048, China

**Keywords:** whole-cell biosensors, polycyclic aromatic hydrocarbons, modification of whole-cell biosensors, synthetic biology, transcription factor

## Abstract

Polycyclic aromatic hydrocarbons (PAHs) and their halogenated derivatives are important targets in environmental monitoring and pollution control because of their persistence, bioaccumulation, and potential carcinogenicity. Reliable strategies for detecting these pollutants remain essential for environmental risk assessment. In recent years, microbial whole-cell biosensors have attracted increasing attention as analytical tools for pollutant detection and toxicity evaluation. These biosensors employ living cells to recognize target compounds and generate measurable signals through endogenous metabolic pathways and transcriptional regulatory networks. As a result, they can reflect biologically relevant responses and operate in complex environmental matrices, making them suitable for in situ monitoring. This review summarises recent advances in whole-cell biosensors for detecting PAHs and their halogenated derivatives. We discuss the design strategies for constructing these whole-cell biosensors and outline their technological development. Recent efforts to improve biosensor performance are also highlighted. Current research trends indicate a shift from optimizing individual genetic components to improving overall system robustness, standardized evaluation, and practical field deployment. These developments provide important insights for designing reliable and engineerable whole-cell biosensing platforms for monitoring PAHs and related pollutants.

## 1. Introduction

Polycyclic aromatic hydrocarbons (PAHs) are a class of organic compounds that contain two or more fused benzene rings. These compounds usually appear as colorless, white, or pale yellow solids. Researchers classify PAHs as persistent toxic pollutants because they are widely distributed in the environment and can also form during food processing [1,2]. Plants and microorganisms can produce PAHs through natural biosynthesis, and natural events such as wildfires in forests and grasslands, as well as volcanic eruptions, also release PAHs into the environment [3]. However, recent reviews have shown that atmospheric PAHs are predominantly derived from anthropogenic sources, particularly from the incomplete combustion of fossil fuels and biomass [4,5]. These activities include incomplete combustion of fossil fuels, improper disposal of industrial chemicals, oil and petrol spills, electricity and heating, tobacco smoke, and emissions from barbecuing [6]. Researchers frequently detect PAHs in a wide range of food products [7]. Studies suggest that PAHs enter the food chain through several pathways, including the uptake of PAHs adsorbed to soil particles by plants and animals and the accumulation of PAHs in aquatic environments. Low-molecular-weight PAHs can also adsorb to food surfaces. In addition, food processing and cooking can further increase PAH contamination, as several studies have reported. Halogenated derivatives of PAHs, including polychlorinated biphenyls (PCBs) and polybrominated diphenyl ethers (PBDEs), are also widely distributed organic pollutants in the worldwide environment. These compounds have attracted increasing attention in environmental science because they exhibit strong environmental persistence, bioaccumulation, and toxic effects on organisms and humans.

Excessive exposure to PAHs increases the risk of several cancers, including pancreatic, gastric, and breast cancers [8]. The United States Environmental Protection Agency (USEPA) has identified 16 PAHs as “priority pollutants” because they can adversely affect aquatic and terrestrial ecosystems. Among these compounds, studies indicate that seven PAHs are either confirmed or suspected carcinogens. This classification highlights the environmental and public health significance of PAHs and emphasizes the need for effective monitoring and management strategies to mitigate their impacts on ecosystems and human health [9]. Consequently, accurate detection and quantification of PAHs in environmental matrices are essential. Monitoring these compounds in food, water, and soil is particularly important because these media are major routes of human exposure. Reliable analytical methods are therefore required to support regulatory control and to reduce the potential health risks associated with exposure to PAHs and their halogenated derivatives.

Conventional analytical techniques for PAHs and their halogenated derivatives rely on multi-step sample preparation and chromatographic–mass spectrometric platforms, which are often labor-intensive, require expensive instrumentation, and demand highly trained operators, limiting their applicability for rapid, on-site monitoring [10,11]. Although GC-MS/MS-based methods can achieve low detection limits for PAHs and PCBs, typically in the range of 0.1–10 µg·L^−1^ for aqueous samples [12,13] and down to 0.02–1.5 µg·kg^−1^ in plant-related matrices [14], and offer a wide linear dynamic range, they still rely on complex laboratory-based analytical workflows that limit their applicability for rapid, on-site monitoring [15]. As contamination of environmental and food matrices by PAHs and PCBs continues to rise, the demand for rapid, on-site, trace-level, and non-destructive detection has grown substantially. In response to this demand, advances in functional material design [16,17], molecular recognition strategies [18], and nanostructured or porous functional materials [19,20] have been developed to improve sensitivity, selectivity, and resistance to interference. Despite these technological advances, both qualitative and quantitative analyses still rely on complex, time-consuming sample-preparation procedures that require substantial amounts of reagents.

**Figure 1:**
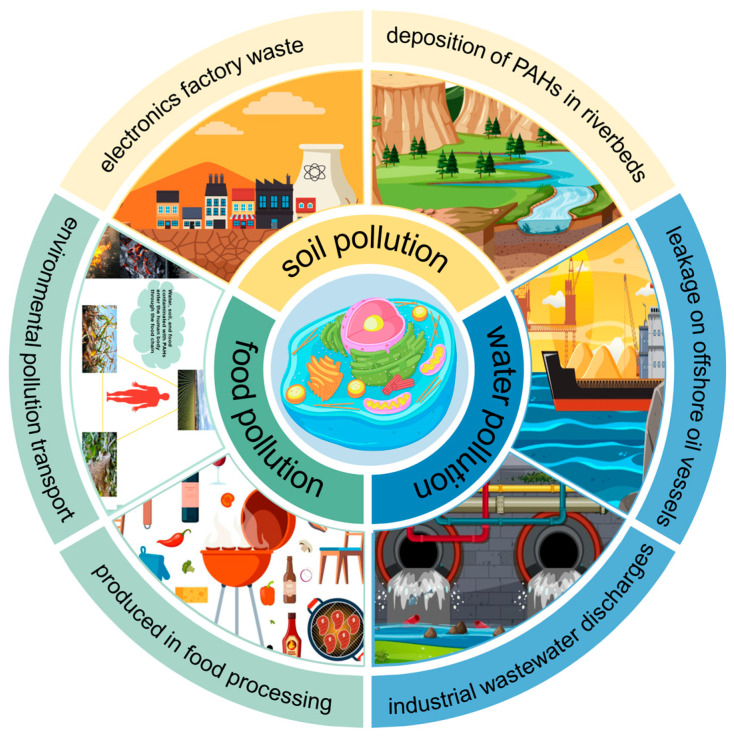
Applications of whole-cell biosensors for detecting PAHs and their halogenated derivatives. The graphical elements were adapted and combined from free images obtained from Vecteezy (https://www.vecteezy.com, accessed on 2 June 2026).

Therefore, it is now necessary to develop such green, efficient, and convenient rapid detection technologies to enable in situ qualitative and quantitative detection of PAHs and their halogenated derivatives. Whole-cell biosensors, which use intact living cells as biological recognition elements, have emerged as a promising alternative. In whole-cell biosensors, chassis cells are the host microorganisms that carry and express engineered genetic circuits, whereas sensing elements are the functional genetic components responsible for target recognition and signal generation, such as promoters, transcription factors, or riboswitches. Compared with other rapid detection approaches, whole-cell biosensors offer several advantages for monitoring PAHs and their halogenated derivatives, including broader detection coverage, high sensitivity and specificity, simplified pre-treatment, reduced analysis time, lower operational costs, portability, and environmental compatibility [21]. By directly responding to the biological effects of contaminants in living systems, whole-cell biosensors enable the assessment of the bioavailable fraction of pollutants and their associated toxicity within complex environmental matrices [22]. As shown in Figure 1, whole-cell biosensors are used to detect PAHs and their halogenated derivatives in diverse environmental contexts.

Although substantial progress has been made in recent studies, a comprehensive review systematically addressing whole-cell biosensors for PAHs and their halogenated derivatives remains lacking. Existing literature rarely offers an integrated perspective on the key functional components and fundamental design strategies underpinning these whole-cell biosensors, and discussions of optimization pathways to address practical application challenges remain limited. This review summarises recent advances in the development of whole-cell biosensors for detecting PAHs and their halogenated derivatives and further discusses the underlying design principles and technological progress. In addition, the major challenges currently confronting this field are examined, and potential future research directions are outlined, with particular emphasis on the importance of interdisciplinary collaboration to accelerate the practical application of whole-cell biosensors in environmental and food contaminant monitoring.

## 2. Overview of the Mechanism of Action of Whole-Cell Biosensors

Whole-cell biosensors are dynamic detection systems that translate biological responses into measurable output signals. Based on the location of target recognition and the pathways of signal transduction, intracellular and extracellular sensing mechanisms have gradually evolved towards coordinated integration. This integrated sensing framework offers new possibilities for detecting membrane-impermeable targets and enables real-time dynamic monitoring. Understanding this coordinated process is therefore important for the rational design and performance optimization of whole-cell biosensors and for improving their reliability in complex sample analysis.

### 2.1. Intracellular Sensing Mechanism

**Figure 2:**
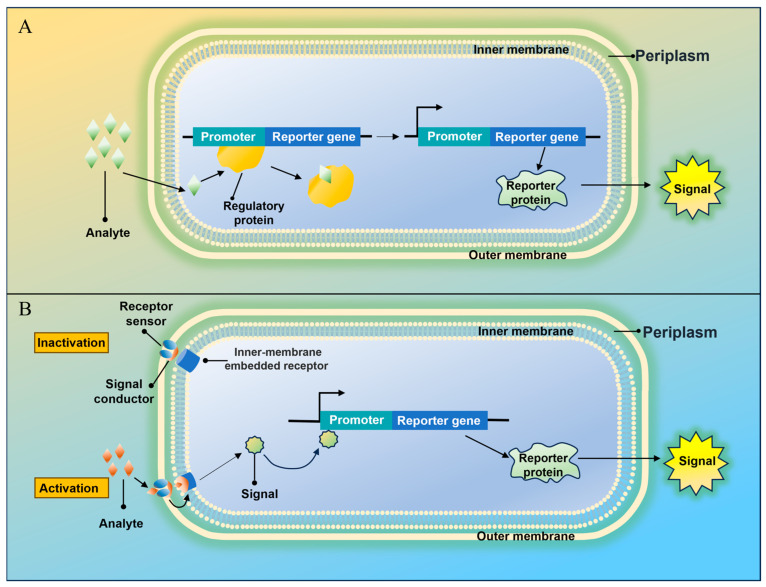
Schematic representation of intracellular and extracellular sensing mechanisms. (**A**) Intracellular sensing mechanisms in whole-cell biosensors. (**B**) Mechanisms of action of whole-cell biosensors outside the cell.

Previous studies show that intracellular sensing in whole-cell biosensors mainly relies on genetically engineered regulatory circuits to achieve target recognition, signal amplification, and quantitative signal output. These systems are typically constructed by genetically modifying living microorganisms, such as bacteria or yeast, to perform specific detection tasks [23]. A typical whole-cell sensing system comprises two key components: a sensing module and a reporter module [24,25]. In the absence of the target molecule, the sensing protein binds to the promoter region, thereby repressing downstream gene expression. When the target compound is present, it interacts with the sensing protein, inducing a conformational change that leads to dissociation from the promoter and relieves transcriptional repression, thereby activating reporter gene expression (Figure 2A).

The intensity of the reporter signal typically correlates with the concentration of the target compound, enabling quantitative detection. In addition to optical outputs, some engineered strains can produce electrochemically active metabolites. These metabolites generate measurable electrical currents when they interact with microelectrodes, thereby linking gene regulation to electrical signal readout and enabling rapid analysis and high-throughput detection [26]. By integrating molecular recognition, signal amplification, and multimodal signal readout within living cells, this sensing strategy provides an important basis for real-time monitoring and online analysis in complex samples.

### 2.2. Extracellular Sensing Mechanism

Building on intracellular sensing, whole-cell biosensors can further expand their detection capability through extracellular sensing. In this strategy, membrane receptors or engineered binding elements capture target molecules outside the cell and transmit signals across the membrane, triggering intracellular responses and thereby broadening the range of detectable targets. Extracellular sensing mainly relies on specific molecular recognition (Figure 2B). These systems use membrane-bound receptors, secreted proteins, or artificially designed binding elements to recognize target molecules and initiate transmembrane signal transduction, such as activating second messengers or two-component regulatory systems. These signaling processes subsequently activate intracellular gene circuits, producing measurable output [27,28].

The interaction between a receptor and a target molecule typically induces a conformational change in the receptor. This structural change converts external physicochemical stimuli into intracellular biochemical signals and regulates downstream enzymatic or transcriptional activity. Signal conversion may occur directly through one-component regulatory systems or indirectly through phosphotransduction in two-component systems. In both cases, extracellular molecular recognition is tightly coupled to intracellular gene circuit regulation. For example, the CadC receptor in bacterial transmembrane signaling systems contains acidic amino acid residues that become protonated at low pH. The receptor functions together with the LysP transporter protein to sense environmental changes and regulate cellular responses [29].

Engineered cells can also secrete aptamers or antibody fragments to capture target molecules in the extracellular environment. The resulting complexes can then be recognised by membrane receptors, which further enhances detection sensitivity. Many hydrophobic pollutants cannot readily cross cell membranes and therefore cannot be efficiently recognised by intracellular sensing systems. This limitation may lead to measured values that are lower than the actual concentrations. To address this problem, researchers combined the luc luminescent gene with the extracellular sensor gene chr1_2466, enabling the luminescent signal to be generated outside the cell [30]. This strategy enables convenient, automated, real-time monitoring and shows strong potential for on-site qualitative and quantitative detection. Although this approach expands the detectable range and improves sensitivity, further studies are still needed to assess detection stability and practical performance in real-world environmental conditions.

## 3. Preparation for the Construction of Whole-Cell Biosensors for PAHs and Their Halogenated Derivatives

Whole-cell biosensors generally consist of three core components: chassis cells, recognition modules, and signal reporter modules. The coordinated function of these elements enables efficient target sensing and signal transduction. Through this integrated design, whole-cell biosensors can achieve sensitive detection of PAHs and their halogenated derivatives.

### 3.1. Common Chassis Cells for Whole-Cell Biosensors of PAHs and Their Halogenated Derivatives

Rational selection and engineering of chassis cells remain essential to improving the environmental robustness and practical applicability of whole-cell biosensors. Chassis cells should exhibit strong ecological adaptability to ensure the biosensor functions reliably in complex, contaminated environments. Table 1 summarises the chassis cells commonly used in related sensor studies, most of which are derived from natural environmental isolates. Native strains often show advantages in specific environmental conditions, but they can also present certain limitations for practical detection. Chassis cells may provide high sensing performance, yet environmental factors can still affect their detection capability. For example, chassis cells used for marine monitoring must withstand high salinity and adapt to temperature fluctuations and other environmental stresses [31].

Species of the genus *Pseudomonas* can degrade a wide range of organic pollutants and have therefore been used as sensing elements for detecting various environmental contaminants [32]. For example, King proposed *Pseudomonas fluorescens* HK44 [33], which carries genes involved in naphthalene degradation, as a chassis cell for constructing a whole-cell biosensor targeting naphthalene. However, *Pseudomonas* species are relatively difficult to manipulate genetically, often have longer growth cycles, and some strains show limited adaptability to changing environmental conditions. These factors limit their broader use in rapid, low-cost detection platforms. To address these limitations, researchers have explored alternative chassis organisms. *Escherichia coli* has gradually become a widely used host for constructing whole-cell biosensors because it is genetically easy to engineer, grows rapidly, and incurs relatively low cultivation costs. For instance, genetically engineered *Escherichia coli*-based whole-cell biosensors have been used to quantify PAH biodegradation and toxicity in various environments, including contaminated soils [34].

Functional microorganisms isolated from specific polluted environments are increasingly regarded as promising chassis cells for constructing whole-cell biosensors because they often exhibit long-term environmental adaptability and specialized metabolic capabilities [35]. For example, *Burkholderia sartisoli* is widely distributed in soil and seawater and is considered harmless to humans. This species can tolerate fluctuations in temperature, pH, and salinity. Using *Burkholderia sartisoli* RP007 as the chassis cell, researchers developed a biosensor for screening marine oil spills [36], which enabled specific detection of several water-soluble compounds. Similarly, the hydrophobic strain *Sphingobium xenophagum* C1, isolated from river sediments contaminated with electronic waste, has evolved a highly hydrophobic cell surface and multiple metal-resistance mechanisms following long-term exposure to high concentrations of PBDEs and heavy metals [37]. The hydrophobic surface of this strain enhances the bioavailability of hydrophobic pollutants and helps overcome the low detection sensitivity often observed in conventional hydrophilic strains. At the same time, its inherent tolerance to pollutants supports stable biosensor signals in complex environments and reduces the need for external additives, such as surfactants. Biosensors constructed from such environmentally adapted microorganisms can leverage the natural ecological robustness of the host cells, thereby enabling sensitive detection of specific pollutants in complex environmental matrices.

**Table 1:** Chassis cells for whole-cell biosensors for PAHs and their halogenated derivatives.

Bacterial Strain	Targeted Compound	Source	Advantage	Disadvantage	LOD	Ref
*Pseudomonas putida* pPG7	Naphthalene	Laboratory genetic engineering construction	Real-time monitoring of hydrophobic contaminants at the single-cell level in complex environments contaminated with biocides directly by gas-phase conductivity.	Does not significantly reduce the detection limit, and the dependence on the kanamycin resistance gene limits its use in a resistance-free environment.	6 μM (aq)/0.6 μM (gas)	[38]
*Burkholderia sartisoli* RP007	Naphthalene, Phenanthrene	Marine water	For complex marine oil pollution monitoring.	Low signal output, complex operation, and limited detection range.	0.17 µM	[36]
					6.7 µM (aq)	[39]
*Pseudomonas fluorescens* HK44	Naphthalene	Wastewater	Harmful organic solvents can be naturally metabolized into non-toxic composites.	Detection accuracy is susceptible to interference, and the detection range of high naphthalene concentrations is limited.	1.2 mg∙L^−1^	[31]
					1 mg∙L^−1^	[40]
*Ralstonia eutropha* ENV307	Polychlorinated Biphenyls	Separation in PCB-contaminated environments	Highly efficient metabolism and sensitive biosensing properties.	Surfactant-dependent and complex assay with low sensitivity.	0.15–1.5 mg∙L^−1^	[41]
Genetically engineered *E. coli*	OH-PCBs	/	Rapid response, matrix tolerance, and cost-effectiveness.	Sensitivity is dependent on exogenous substrates due to significant compound differences, time-consuming assays, and potential cross-reactivity.	5.0 × 10^−8^ M (serum)	[42]
*Sphingobium xenophagum* C1	PBDEs	E-waste contaminated river sediment	Excellent ecological adaptation of natural hydrophobic chassis cells in complex contaminated environments.	The genetic manipulation system is immature and relies on specific outer membrane receptor proteins, limiting the generalisability of practical applications.	0.01 µM	[30]

### 3.2. Sensing Elements for Whole-Cell Biosensors for PAHs and Their Halogenated Derivatives

The construction of whole-cell biosensors for pollutant detection relies on sensing elements that specifically recognize target molecules. In studies targeting PAHs, polychlorinated biphenyls, and polybrominated diphenyl ethers, researchers have gradually expanded the types of sensing elements used in biosensor design. These elements now range from intracellular transcription factors to membrane-associated sensor proteins, enabling broader molecular recognition and improved sensing performance.

**Figure 3:**
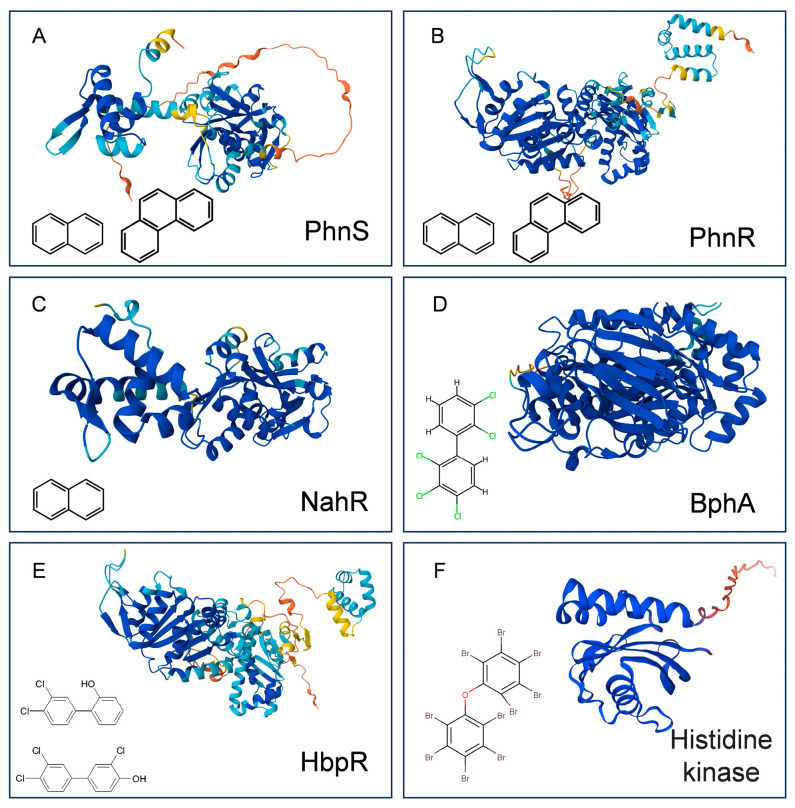
Sensing elements for whole-cell biosensors for PAHs and their halogenated derivatives. Protein structure obtained from the AlphaFold Protein Structure Database, refs. [50,51]. (**A**) PhnS. (**B**) PhnR. (**C**) NahR. (**D**) BphA. (**E**) HbpR. (**F**) Histidine kinase.

Detection of PAHs in whole-cell biosensors often relies on the specific recognition ability of transcription factors. Among the most widely studied regulators are PhnR and PhnS, which are commonly used for phenanthrene detection (Figure 3A,B), and NahR, which is frequently used for naphthalene sensing (Figure 3C). PhnS and PhnR were originally identified in *Burkholderia sartisoli* RP007 [43]. PhnS belongs to the LysR-type transcriptional regulator family and activates the phn gene cluster in response to phenanthrene. In contrast, PhnR is a σ^54^-dependent positive regulator in the NtrC family. These two regulators coordinately control the degradation pathways of phenanthrene and naphthalene. The transcription factor NahR, derived from *Pseudomonas putida* G7, also belongs to the LysR family [44,45], but its regulatory mechanism has been more clearly characterized. In the presence of salicylic acid, the NahR protein binds to a conserved DNA sequence upstream of the *nah* operon promoter and activates transcription of the upper naphthalene degradation pathway. Salicylic acid then acts as an inducer, further stimulating the lower degradation pathway and ultimately enabling the reporter strain to produce a quantifiable luminescent signal.

Detection of polychlorinated biphenyls (PCBs) and their derivatives often requires more sophisticated sensing element design. The biodegradation of PCBs mainly proceeds through the biphenyl operon-mediated catabolic pathway, in which the enzyme biphenyl dioxygenase BphA (Figure 3D) catalyzes the initial oxidation step and largely determines the substrate recognition range [46]. Researchers isolated the *orf0-bphA1* gene cluster from *Ralstonia eutropha* ENV307 [47,48]. However, the upstream *orf0* gene shows low homology to known transcriptional regulators, suggesting a potentially distinct regulatory mechanism [42]. For hydroxylated polychlorinated biphenyls, the transcription factor HbpR (Figure 3E), derived from *Pseudomonas azelaica* HBP1, has been identified as a LysR-family regulator capable of specifically recognising hydroxylated compounds with a biphenyl backbone. In the absence of an inducer, HbpR binds to the promoter region and represses transcription. Upon binding to the inducer molecule, HbpR undergoes a conformational change that relieves repression and activates downstream gene expression. Based on this regulatory mechanism, HbpR has been widely used to construct whole-cell biosensors for detecting hydroxylated biphenyl compounds.

Although transcription factor-based sensing systems have achieved considerable success in whole-cell biosensor detection, these elements operate intracellularly and therefore rely on efficient transmembrane transport of target molecules. For highly hydrophobic persistent organic pollutants, this transport process can become the rate-limiting step, leading to delayed responses or reduced detection sensitivity. To address this limitation, recent studies have begun exploring sensing elements on the cell surface. For example, Chen and colleagues [30] compared the transcriptomic responses of the hydrophobic strain *Sphingobium xenophagum* C1 and its variants under exposure to decabromodiphenyl ether and identified a membrane-associated protein, Chr1_2466 (Figure 3F). This protein contains a Cache domain and is localized on the outer membrane, enabling extracellular recognition of polybrominated diphenyl ethers.

Unlike transcription factors that rely on intracellular degradation pathways, Chr1_2466 directly recognizes decabromodiphenyl ether and its lower-brominated homologs, yet shows no response to 29 potential interferents, including polychlorinated biphenyls and phenolic compounds [49]. The binding affinity of this protein increases with the number of bromine atoms in the target molecules. Interestingly, its localization at the membrane surface enables real-time monitoring of target molecules via fluorescence without the need for cell lysis. This feature significantly improves detection convenience and provides a promising strategy for the in situ monitoring of hydrophobic organic pollutants.

### 3.3. Commonly Used Reporter Elements for Whole-Cell Biosensors of PAHs and Their Halogenated Derivatives

During the construction of whole-cell biosensors, the promoter responsive to the target analyte is typically fused to an appropriate reporter gene. The expressed reporter protein can generate detectable signals in various forms, including colorimetric, fluorescent, bioluminescent, chemiluminescent, or electrochemical outputs [49]. Different reporting platforms exhibit significant differences in signal generation mechanisms, metabolic dependence, and field applicability, and their selection directly influences the biosensor’s detection performance and practical value [52]. Table 2 summarises commonly used reporter elements for constructing whole-cell biosensors targeting PAHs and their halogenated derivatives. Owing to their favourable catalytic properties, efficient light emission, and broad applications in bioluminescent systems, fluorescent proteins and luciferases have become major research foci in biosensing. Green fluorescent protein, originally isolated by Osamu Shimomura from *Aequorea victoria*, derives its fluorescence from a chromophore formed autocatalytically by the Ser65-Tyr66-Gly67 tripeptide within the protein [53,54]. The principal advantages of the GFP system lie in its independence from exogenous substrates, low cytotoxicity, and suitability for real-time imaging in living cells, while maintaining structural stability over a relatively broad pH range. However, chromophore maturation requires time, which may delay signal output and reduce temporal resolution. In addition, background fluorescence and light scattering, which are commonly present in environmental samples, can markedly decrease the signal-to-noise ratio, thereby limiting sensitivity for trace pollutant detection [55]. Consequently, GFP is more suitable for mechanistic investigations or cellular-level response analyses, whereas it is generally less advantageous for monitoring low-concentration environmental contaminants.

In contrast, bioluminescent systems based on the *lux* genes exhibit superior performance in environmental detection due to their low background noise and high signal sensitivity. The *lux* gene cluster typically comprises *luxICDABE* together with the regulatory element *luxR* [56]. Among them, *luxAB* encodes the heterodimeric luciferase, while *luxCDE* participates in the endogenous regeneration of the fatty aldehyde substrate. When the complete *luxABCDE* cluster is employed, cells can achieve continuous, substrate-self-sufficient luminescence without exogenous substrate addition, enabling in situ and online monitoring strategies [57]. The luminescent reaction depends on molecular oxygen, oxidising fatty aldehydes to the corresponding carboxylic acids and emitting visible light [58], with emission intensity closely correlated with cellular metabolic activity and transcriptional levels. Because the luminescence process is highly dependent on ATP supply and aerobic metabolic conditions, signal attenuation readily occurs under nutrient-limited or hypoxic environments in whole-cell biosensors [59,60], which imposes potential constraints for applications in sediments or highly contaminated microenvironments.

Firefly luciferase is derived from the North American firefly *Photinus pyralis* [61,62]. Its encoding gene, *luc*, has been widely applied in bioluminescent reporter systems because of its high catalytic efficiency and strong light emission. In the presence of ATP, Mg^2+^, and oxygen, this enzyme catalyses the oxidation of luciferin, producing a high quantum yield and intense, instantaneous light output, making it suitable for high-precision quantitative analysis. In the construction of hydrophobic whole-cell biosensors, luciferase catalyses the oxidation of the substrate furimazine, generating a bioluminescent signal proportional to the concentration of PBDEs [30]. However, firefly luciferase shows relatively limited thermal stability, and its sustained performance under complex environmental conditions is somewhat constrained. In addition, the requirement for exogenous furimazine increases detection costs.

**Table 2:** Reporter elements used in whole-cell biosensors for PAHs and their halogenated derivatives.

Reporter Component	Reporter Gene	Output Signal Type	Targeted Compound	Response Time	Functional Lifetime	Source	Ref
Green fluorescent protein	*gfp* gene	Green fluorescence	Phenanthrene	24–48 h	~2–3 days	The jellyfish *Aequorea victoria*	[39]
*Bioluminescent luciferase*	*lux* gene/*luxCDABE*	Bioluminescence	Naphthalene	4–6 h	~80 h	Bioluminescent marine bacteria	[38]
			Naphthalene	54 min	not reported		[63]
			Pyrene, Beno[a]pyrene	24 h	≥8 weeks (4 °C storage)	*Photorhabdus luminescens*	[64]
			Phenanthrene	6 days	not reported	*Pseudomonas putida*	[23]
			PCBs	1–3 h	not reported	*Vibrio fischeri*	[41]
	*luxAB*	Bioluminescence	OH-PCBs	4 h	not reported	*Vibrio fischeri*	[42]
*Cell-killing* gene	*gef* gene	Decrease in fluorescence intensity	Peno[a]pyrene	24 h	not reported	Gram-negative bacteria	[34]
HiBiT-Luc fusion system	HiBiT peptide label and luciferase	Fluorescent signal	PBDEs	8 h	not reported	Synthetic 11 peptides and Photinus pyralis	[30]

## 4. Preliminary Construction of Whole-Cell Biosensors for PAHs and Their Halogenated Derivatives

Traditional transcription- and regulation-based whole-cell biosensors were the core technological paradigm in early studies monitoring the bioavailability of PAHs and their halogenated derivatives. Their design relies on inducible transcriptional regulators derived from microbial aromatic hydrocarbon degradation pathways. In these systems, pollutant-responsive promoters are transcriptionally fused with reporter genes, enabling biological signals generated upon pollutant recognition to be converted into quantifiable optical outputs.

### 4.1. Early Biosensor Construction Strategies Based on Genetic Circuits

During the early development of whole-cell biosensors, transcriptionally regulated genetic circuits established the technical foundation of the field. Burlage and colleagues developed a *lux*-based real-time reporting system and, for the first time, transcriptionally fused the *P*_nah_ promoter from the NAH7 plasmid with the *luxCDABE* gene cluster to construct plasmid pUTK9, enabling real-time and noninvasive monitoring of naphthalene catabolism [65]. In this design, a pollutant-responsive promoter drives the expression of the *luxCDABE* bioluminescent genes, thereby directly coupling metabolic activity to optical signal output. Consequently, luminescence intensity dynamically reflects the degradation status of substrates such as naphthalene. This metabolism-driven signal transduction architecture represents a transition from endpoint detection to real-time monitoring and provides an operational framework for evaluating exposure to environmental pollutants. However, these early genetic circuit fusions lacked insulation and modular design, which made them susceptible to effects from construct orientation and genomic insertion sites and could compromise system stability. Therefore, effective circuit design should ensure that functional genetic modules remain insulated from host regulatory backgrounds and other introduced genetic elements, thereby preventing signal interference or regulatory crosstalk and maintaining the biosensing circuit’s independence and stability.

King and colleagues subsequently constructed the reporter plasmid pUTK21 and developed the engineered strains 5RL and HK44 as whole-cell biosensors for real-time monitoring of naphthalene exposure and biodegradation [33]. This system responds rapidly, within 15 min of induction by naphthalene or salicylic acid, and the luminescence intensity remains synchronized with naphthalene degradation dynamics. However, practical applications still face several challenges. Limited specificity restricts the broader applicability of this system and highlights the need for highly specific recognition modules in real-world deployment.

### 4.2. Metabolically Coupled Whole-Cell Biosensors: Strategies Centered on Bioavailability

From the perspective of environmental risk assessment, the bioavailability of pollutants is often more ecologically relevant than their total concentration. Early whole-cell biosensors were therefore frequently designed to couple pollutant uptake and metabolic processes to signal generation, thereby converting metabolic flux into detectable outputs. This strategy enables dynamic evaluation of the bioavailable fraction of target compounds and, in principle, provides a more realistic representation of pollutant accessibility to microbial communities. However, the performance of such systems is strongly influenced by the physiological state of the host cells and by environmental conditions.

Represented by the naphthalene-responsive system constructed by Heitzer and colleagues [66], a whole-cell biosensing platform based on the nahG-luxCDABE fusion protein was developed to quantitatively assess the bioavailability of naphthalene and salicylate in environmental samples. Their results demonstrated that cells in the exponential growth phase exhibited a stronger and more reliable bioluminescence response than carbon-starved cells, while also reducing interference from non-target substrates. The higher basal metabolic activity and increased reporter output per unit biomass indicate an intrinsic biological signal amplification effect within the system. However, the study also showed limited reproducibility, with significant variations in calibration curve slopes across independent experiments. In complex matrices such as soil slurry, signal attenuation occurred due to adsorption of target compounds and optical quenching, resulting in a marked reduction in detection efficiency compared with aqueous samples. These matrix-dependent effects pose challenges for field deployment and long-term operational stability.

Furthermore, the engineered strain *Pseudomonas fluorescens* HK44 carrying the nahG-luxCDABE reporter gene system was immobilized in a strontium alginate matrix [67]. The immobilized cells stored at 4 °C maintained viability during short-term storage, and the immobilization process enhanced cell retention, thereby enabling stable signal generation under continuous-flow conditions. A dual-channel HPLC pump system was employed to precisely mix the maintenance medium and the sample stream, thereby providing stable and reproducible hydrodynamic conditions. In addition, a flow-through configuration with an integrated optical probe was constructed, enabling real-time bioluminescence monitoring during continuous operation. However, temporal variations in cellular activity and diffusion limitations within the immobilization matrix may lead to signal drift, potentially affecting baseline stability. Therefore, periodic recalibration is required in practical applications. In addition, field deployment may be constrained by system maintenance requirements and the need for strictly controlled flow conditions.

Similarly, Tecon et al. constructed a phenanthrene-responsive fluorescent biosensor based on the PhnR–phnS regulatory system from *Burkholderia* sp. RP007 by fusing the phnS promoter to enhanced GFP, enabling amplified fluorescence output through transcriptional activation during phenanthrene metabolism [39]. The generated signal was directly correlated with pollutant bioavailability, providing practical relevance for ecological risk assessment, and metabolic activity could be maintained through supplementation with auxiliary carbon sources to improve signal stability. In essence, this system depends on multiple biological processes, including pollutant uptake, metabolic transformation, and subsequent transcription and translation, resulting in an inherently multi-step response with an unavoidable temporal delay. At the same time, reliance on a single fluorescent output limits signal amplification capacity, while output intensity is highly sensitive to variations in host metabolic state and environmental conditions. In real environmental matrices, coexisting aromatic compounds, unknown inhibitors, and matrix-associated toxicity can further distort signal output and reduce cellular viability, thereby limiting quantitative accuracy. These inherent constraints restrict the robustness and field applicability of transcriptional regulation-based biosensors in complex real-world settings.

### 4.3. Application Expansion of Traditional Genetic Circuits in Pollutant Detection

Within the framework of metabolically coupled genetic circuits, whole-cell biosensors were further developed to respond to low solubility and hydrophobic organic pollutants, thereby enabling indirect characterisation of their bioavailability. Layton and colleagues constructed a whole-cell biosensor centred on *Ralstonia eutropha* ENV307 carrying pUTK60, in which the regulatory module of the biphenyl degradation pathway, *orf0-bphA1*, was fused with the *lux* gene cluster, allowing induction signals from polychlorinated biphenyls to be converted into measurable luminescent outputs [41]. To address the low solubility and limited environmental availability of PCBs, the system incorporated nonionic surfactants to enhance substrate solubilization and mass-transfer efficiency, thereby significantly improving detection sensitivity, with a minimum detection limit of 0.15 mg∙L^−1^. Experimental results demonstrated that biphenyl, monochlorobiphenyl, and Aroclor 1242 could induce a three to fourfold increase in luminescence, indicating the potential applicability of this system in complex environmental samples. Although this study extended the application boundaries of traditional genetic circuits for detecting hydrophobic persistent pollutants, its strong dependence on mass-transfer efficiency and metabolic balance also highlights substantial potential to improve engineering controllability in whole-cell biosensing strategies. However, the reported detection limits and induction factors are dependent on surfactant-assisted solubilization of hydrophobic substrates, introducing uncertainty in environmental reproducibility.

## 5. Optimization Strategies for Whole-Cell Biosensor Systems Targeting PAHs and Their Halogenated Derivatives, Driven by Structural Constraints

Early whole-cell biosensors for PAHs and their halogenated derivatives were primarily constructed based on natural aromatic hydrocarbon degradation regulatory networks. Their sensing performance was constrained by several inherent structural bottlenecks, including insufficient molecular recognition specificity, limited mass transfer efficiency, inefficient metabolic coupling, and the metabolic burden imposed on host cells. In recent years, optimization strategies have gradually shifted from modifying individual components to system-level reconstruction, aiming to improve sensing performance across multiple functional layers, including molecular regulation, interfacial mass transfer, metabolic flux distribution, and chassis homeostasis.

### 5.1. Optimization at the Level of Molecular Recognition and Transcriptional Regulation

The precision of molecular recognition determines the selectivity and sensitivity of whole-cell biosensors. Structural optimization of natural aromatic-responsive transcription factors has become an important strategy for improving recognition efficiency. For example, Shin and colleagues performed site-directed mutagenesis in the inducer-binding region and oligomerization domain of the NahR protein to improve the sensitivity of a microbial biosensor for salicylate detection [68]. Among the generated variants, the N169C and R248Q mutants significantly altered the transcriptional response, achieving up to a 50-fold increase in luminescence under optimized inducer concentrations. This improvement is mainly attributed to changes in inducer-binding affinity and alterations in regulatory conformational dynamics, which enhance transcriptional activation efficiency. However, enhanced signal output does not necessarily correspond to improved analytical performance, as it may be accompanied by increased background activity or reduced specificity, particularly in mutants with broadened ligand recognition profiles. Therefore, although rational mutagenesis can significantly improve sensitivity, maintaining specificity and response stability remain key challenges in biosensor optimization.

In addition to transcription factor engineering, promoter optimisation is also used to enhance the strength of the response. The metabolites of polychlorinated biphenyls (PCBs), such as hydroxylated PCBs (OH-PCBs), have been identified as environmental contaminants. Various studies have shown that some OH-PCBs can potentially contribute to health problems. In addition to transcription factor engineering, promoter optimization has also been employed to enhance response intensity. Turner and colleagues [42] utilized an hbpR-regulated promoter that specifically recognizes hydroxylated biphenyl skeletons, thereby excluding interference from parent PCBs and non-hydroxylated analogs. On this basis, they constructed a genetically engineered whole-cell biosensor integrating the HbpR regulatory protein with the *luxAB* luciferase reporter genes, thereby improving detection stability and sensitivity. This biosensor overcame the dependence of conventional chromatographic mass spectrometric techniques on extensive sample purification, enabling direct detection of OH-PCBs and multiple OH-PCBs isomers in complex matrices such as human serum without complicated pretreatment. This advancement extended biosensing technology from laboratory-based research to metabolite toxicity monitoring, providing a high-throughput, rapid, and cost-effective on-site screening tool for evaluating the biological activity of persistent organic pollutants and supporting clinical toxicology studies. However, the application of this biosensor is still largely confined to controlled experimental settings or spiked samples, and its performance in truly heterogeneous environmental matrices has yet to be fully validated. In addition, the need for optimized assay conditions and external sample processing may limit its direct applicability for routine field deployment. To further enhance downstream metabolic gene expression and shorten response time, Sun and colleagues proposed replacing the native nahR regulatory system with the constitutive strong promoter Ptet to directly drive high-level expression of the *nahAD* gene cluster [69]. Although such strong driving strategies may increase basal expression and metabolic burden, optimization at the transcriptional regulation level has demonstrated significant effectiveness in strengthening signal output.

### 5.2. Optimization of Mass Transfer and Interfacial Engineering

The low aqueous solubility and slow diffusion rate of PAHs such as naphthalene limit their contact efficiency with cells. These physicochemical properties result in insufficient sensitivity of conventional whole-cell biosensors when detecting low concentrations of hydrophobic organic pollutants, making it difficult to reach detection limits required for practical environmental monitoring. Optimization of interfacial mass transfer has therefore become a critical breakthrough strategy. Werlen and colleagues developed a gas phase detection system that exploited the gas–water partitioning characteristics of PAHs to significantly enhance diffusion rates [70], thereby improving sensitivity at low concentrations. The research group immobilized the engineered *Pseudomonas putida* pPG7-JAMA21 strain onto the surface of a nylon membrane with a pore size of 0.45 μm, thereby reducing mass-transfer resistance in the aqueous boundary layer and preventing signal delays caused by diffusion limitations around suspended cells. Although this approach offers clear theoretical advantages in mass transfer kinetics, its stability and level of standardization in complex environmental samples still require further validation. The gas-phase configuration not only enhances sensitivity but also more realistically simulates microorganisms’ natural exposure to pollutants at the soil–air interface, thereby increasing the environmental relevance of the detection results.

During the detection of target chemicals by whole-cell biosensors, the cell membrane can act as a barrier [71]. Under such conditions, sensitivity toward the target compound largely depends on its uptake efficiency. Therefore, increased membrane permeability or enhanced cellular uptake of the analyte generally leads to improved detection sensitivity [72]. The introduction of transmembrane sensing proteins has provided a new strategy for recognizing hydrophobic pollutants. Engineering the cell–surface interface helps overcome mass-transfer limitations for highly hydrophobic pollutants in real environmental samples. For example, a novel Cache domain containing a transmembrane receptor identified in tolerant strains has been shown to specifically recognize the brominated sites of PBDEs [30], providing a biological basis for improving pollutant recognition at the cell–environment interface. By coupling this receptor with a nano-fluorescent labeling system and exploiting the intrinsic hydrophobicity of the host cell surface as a natural enrichment interface, PBDE molecules can be more effectively accumulated near the sensing interface, thereby increasing their local contact probability with the recognition element. However, this interface-driven enrichment remains closely dependent on both cell-surface properties and environmental matrix composition; therefore, its performance may vary across sample types and is not always readily reproducible under complex conditions.

### 5.3. Optimization of Metabolic Efficiency and Signal Amplification Mechanisms

Conventional PAH biosensors typically rely on the metabolic conversion of pollutants to generate signal molecules that subsequently trigger reporter expression, meaning that metabolic flux directly determines the efficiency of signal output. To enhance responsiveness to low-concentration pollutants, researchers have optimized signal generation rates through pathway substitution and metabolic module reconstruction. For instance, overexpression of key enzymes in the naphthalene degradation pathway has been used to improve salicylic acid production efficiency, as the gene cluster from *Pseudomonas putida* encodes enzymes that specifically catalyze naphthalene metabolism via the salicylate pathway without significantly affecting other organic substrates [69]. In addition, modular sensing architectures based on standardized signaling systems such as salAR–lux have enabled the flexible construction of biosensors for different PAHs by replacing upstream metabolic modules, thereby improving system adaptability and multiplexing capability.

However, these metabolic optimization strategies often rely on increased heterologous gene expression, which can impose a metabolic burden on host cells and disrupt cellular resource allocation. Such imbalances may lead to reduced growth fitness, variability in signals, or long-term instability, particularly under nutrient-limited or environmentally variable conditions. Therefore, while metabolic optimization can significantly enhance signal amplification capacity, its effectiveness is ultimately constrained by the need to maintain host metabolic homeostasis. Future design strategies should therefore focus on balancing pathway efficiency with cellular resource management to ensure stable, robust, and reproducible biosensing performance in complex environmental applications.

### 5.4. Optimization of Chassis Cell Homeostasis

The chassis cell functions not only as the execution unit for signal generation but also as a major constraint in whole-cell biosensor systems. High-level expression and extensive metabolic reconstruction inevitably increase resource consumption and physiological stress, resulting in signal drift or growth limitation. Therefore, the objective of chassis engineering is not simply to maximize expression levels, but to establish a homeostatic balance between low metabolic burden and high signal output. For example, *Acinetobacter* ADPWH_lux with low background luminescence has been employed as a host strain [73]. This strain exhibits high compatibility with heterologous expression of the *nahAD* genes [69], and its plasmid demonstrates strong stability, maintaining a survival rate above 90 percent for 72 h even in the absence of antibiotic selection, thereby enabling long-term stable operation of the biosensor. In addition, optimizing gene integration strategies and constructing negative control systems can further reduce endogenous interference. At present, most designs still rely on static expression modes and lack dynamic feedback regulation mechanisms. Future chassis optimization should incorporate resource-allocation sensing and adaptive regulatory networks, enabling the sensing system to achieve environment-driven homeostatic self-regulation and improved long-term stability.

## 6. Design Trends and Field-Oriented Application Prospects of Whole-Cell Biosensors for PAHs and Their Halogenated Derivatives

### 6.1. From Empirical Assembly to Programmable Architectures: A Systematic Upgrade Path for Whole-Cell Biosensors

Although whole-cell biosensors have demonstrated satisfactory detection performance for PAHs and their halogenated derivatives under controlled laboratory conditions, their translation to real environmental monitoring scenarios remains constrained [74]. The primary challenges are not limited to deficiencies in individual performance parameters but arise from structural limitations, including constraints imposed by native metabolic networks, limited plasticity of regulatory elements, and insufficient system stability under complex environmental interference.

On-site real-time detection requires these biosensors to operate under more stringent and variable conditions, placing higher demands on robustness, adaptability, and signal reliability. Consequently, considerable room remains for optimization in the design of whole-cell biosensors targeting PAHs and their halogenated derivatives. In response to these challenges, the development paradigm for whole-cell biosensors is shifting from empirical circuit assembly to programmable, modular system architectures. This transition aims to decouple and reconfigure key functional layers, including molecular recognition, signal processing, and field-deployment capability, thereby enabling more rational design, improved environmental resilience, and scalable application potential.

#### 6.1.1. Reconstruction of the Recognition Layer to Overcome Metabolic Constraints

Conventional whole-cell biosensors primarily rely on native regulatory elements for pollutant recognition. However, these transcriptional regulators evolved to support metabolic adaptation rather than analytical specificity. As a result, they often exhibit cross-reactivity, limited dynamic range, and insufficient selectivity, which restrict their applicability in complex environmental matrices. Recent advances have sought to overcome these limitations through synthetic biology strategies, including directed evolution, rational design, and machine learning-assisted mutational prediction [75]. Engineering ligand-binding domains at the molecular level also enhances binding specificity and expands the dynamic response window. When coupled with high-throughput fluorescence screening platforms and multi-omics analyses, including transcriptomics, proteomics, and metabolic flux analysis, the global impact of regulatory network rewiring on signal output can be systematically evaluated.

Nevertheless, regulatory protein engineering is frequently accompanied by increased metabolic burden and perturbation of endogenous networks. Long-term stability and genetic escape frequency remain insufficiently characterized, particularly under field-relevant conditions. Moreover, multi-input logic-gate circuits enable the integration of environmental parameters, such as pH and temperature, with target analytes, thereby improving contextual accuracy [22]. However, increased circuit complexity may introduce response delays and compromise system robustness. Therefore, future design should move beyond optimizing single performance metrics and instead pursue a holistic balance between selectivity, programmability, and long-term stability. The development of robust recognition modules and resilient regulatory architectures remains a central research priority.

#### 6.1.2. Toward Multimodal Construction of the Signal Output Layer

The reporter system not only determines detection sensitivity but also directly influences field applicability. The use of multicolor fluorescent proteins enables multiplexed detection within single cells; however, spectral crosstalk, photobleaching, and reliance on optical instrumentation limit their robustness under field conditions. In addition, dual-output sensing architectures that integrate fluorescent and bioluminescent reporters enable multi-channel signal generation. Algorithm-assisted cross-validation of the two output signals significantly reduces false-positive responses arising from interference affecting a single reporting pathway. For example, Wang et al. developed the *Streptomyces*-based sensor WHY03 [76], integrating transcriptomics-screened promoters to achieve efficient detection of glycopeptide antibiotics. Several studies have demonstrated relatively broad dynamic ranges using such systems. Current efforts predominantly focus on enhancing signal intensity, whereas systematic optimisation of signal reliability, anti-interference capacity, and long-term operational cost remains comparatively underexplored. Future reporter module design should prioritise an integrated balance among metabolic burden, signal robustness, and field adaptability, rather than pursuing signal amplification alone.

#### 6.1.3. From Living-Cell Platforms to Cell-Free Systems

Signal output from whole-cell biosensors is inherently influenced by cellular growth phase and metabolic state. Variations in gene expression under different physiological conditions significantly reduce detection reproducibility, and whether system performance can remain stable over prolonged operation remains an unresolved challenge, particularly in field applications. Cell-free protein synthesis-based biosensing platforms offer a promising alternative [77,78]. By reconstructing transcription and translation machinery in vitro, cell-free systems eliminate growth-dependent fluctuations and reduce biosafety concerns associated with live microorganisms. These platforms utilise enzymatic components derived from cell lysates or synthetically assembled functional modules to complete target-responsive transcription and translation in the absence of living cells [79]. Such decoupling from cellular viability enhances controllability and improves batch-to-batch consistency.

In parallel, microfluidic platforms provide spatial confinement and precise environmental control, thereby enhancing operational stability and device miniaturisation. Immobilised yeast or bacterial systems have already been explored for pollutant detection in wastewater, demonstrating the feasibility of semi-contained deployment strategies [80,81]. Future development should integrate engineered recognition modules, multimodal signal output architectures, and hardware-assisted stabilisation frameworks to establish truly deployable biosensing platforms. Compared with incremental improvements in individual performance metrics, rigorous evaluation of long-term stability and standardised validation protocols will ultimately determine the translational potential of next-generation whole-cell and cell-free biosensor systems.

### 6.2. Practical Application Prospects of Whole-Cell Biosensors

In detecting PAHs and their halogenated derivatives, whole-cell biosensors have made substantial progress at the laboratory scale. However, their true value lies in whether they can be translated into deployable technologies. Despite advances in sensitivity and specificity under controlled conditions, practical implementation requires robustness and operational simplicity.

As biosensor research frameworks continue to mature, the focus has gradually shifted from fundamental mechanistic exploration toward translational development. Increasing attention is being directed to portable device integration, on-site environmental monitoring, and applications in food safety assessment. This transition reflects a broader recognition that analytical performance alone is insufficient; scalable fabrication, storage stability, user-friendly interfaces, and cost-effectiveness are equally critical for real-world adoption. Accordingly, future efforts must bridge the gap between laboratory validation and field detection by integrating biological engineering with hardware design, standardised calibration protocols, and scenario-specific performance evaluation.

#### 6.2.1. Miniaturised Sensing Systems for Field Detection

**Figure 4:**
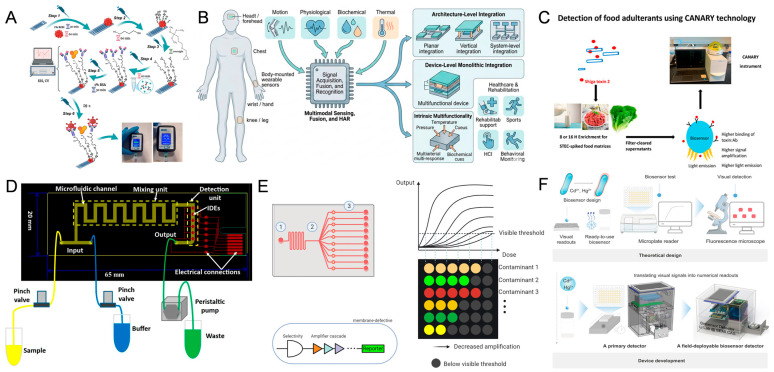
Prospects for whole-cell biosensors for PAHs and their halogenated derivatives. (**A**) Schematic of a portable biosensor enabling highly sensitive detection of the Parkinson’s disease biomarker DJ-1. Adapted from ref. [83]. (**B**) Illustration of wearable multifunctional sensors for health monitoring applications. Adapted from ref. [85]. (**C**) Schematic of a portable cell-based sensing assay for rapid detection of Shiga toxin (Stx). Adapted from ref. [87]. (**D**) Schematic diagram of a portable automated detection system integrated with a microfluidic platform. Adapted from ref. [89]. (**E**) Scheme as well as optimization of microfluidic-based whole-cell biosensors for simultaneous detection of multiple pollutant types and levels. Adapted from ref. [81]. (**F**) Portable device based on whole-cell biosensors and its application in environmental monitoring. Adapted from ref. [91].

In field detection scenarios, sensitivity alone is insufficient; system miniaturisation and operational simplicity are equally critical. Recent advances in portable analytical devices illustrate the feasibility of integrating biological recognition with compact hardware platforms. For instance, a glucose meter-based platform combined with the CRISPR-Cas12a system has been developed to build a portable biosensor that detects kanamycin at concentrations as low as 1 pM in water samples, demonstrating the potential of coupling nucleic acid amplification mechanisms with user-friendly devices [82]. A portable immunopotentiometric sensor was developed by combining AuNP-modified SPCE with GPTMS, enabling highly sensitive detection of the Parkinson’s disease biomarker DJ-1, with a limit of detection as low as 0.00059 ng∙mL^−1^ (Figure 4A) [83]. In another example, Shen and colleagues reported a flexible and highly sensitive wearable lactate biosensor for real-time monitoring of lactate levels in human sweat [84], highlighting the promise of continuous biochemical sensing in accessible biofluids. Wearable multifunctional portable sensors have demonstrated their potential applications in medical rehabilitation, sports science, and human–computer interaction (Figure 4B) [85].

The central objective of such systems is to enable real-time and continuous quantification of target analytes in readily obtainable samples. However, their applicability to complex environmental pollutants, particularly low-solubility polycyclic aromatic hydrocarbons, remains insufficiently validated. For hydrophobic contaminants, limitations in sample pretreatment and bioavailability may become critical bottlenecks for portable, real-time detection. Therefore, miniaturisation strategies should be aligned with the physicochemical characteristics of target analytes rather than driven solely by device size reduction. Effective field-oriented design requires coordinated optimisation of sampling interfaces, biological recognition modules, and hardware integration to ensure analytical reliability under realistic environmental conditions.

#### 6.2.2. Feasibility in Complex Food Matrices

Food safety has become a global concern, and monitoring strategies require sensors that maintain stable performance in complex matrices characterised by high protein and lipid levels and multiple interfering components. For example, a high-throughput detection system constructed using cardiomyocytes integrated with microelectrode arrays can reflect the overall physiological effects of ion-channel toxins [86]. Pathogenic Shiga toxin-producing *Escherichia coli* (STEC) causes severe foodborne diseases. Researchers developed a rapid and portable cell-based sensing assay for detecting Shiga toxin (Stx), which was successfully applied to sensitively detect STEC contamination in food samples such as milk, lettuce, and beef (Figure 4C) [87]. Compared with other sensing platforms, whole-cell biosensors have a distinctive advantage: living cells serve as natural sensing units, enabling direct assessment of the integrated toxicological impact of contaminants. This feature has demonstrated particular value in the monitoring of toxins [7].

Quality control also represents a critical dimension of food safety assessment. In response to excessive heavy metal contamination in food products, several biosensing strategies based on aptamers’ specific affinity for metal ions have been developed [88]. Paper-based microfluidic platforms that employ aptamer-mediated recognition enable simultaneous detection of multiple heavy metal ions while significantly reducing analytical costs. Driven by the need to monitor pesticide residues, a portable automated detection system integrated into a PDMS microfluidic chip was developed, enabling highly sensitive and selective simultaneous detection of tebuconazole and λ-cyhalothrin (Figure 4D) [89]. In addition, the cell-free paper-based putrescine sensor developed by Selim and colleagues further validated the feasibility of low-cost platforms for food quality control [90]. Future biosensors targeting PAHs and their halogenated derivatives can draw on these design principles by integrating paper-based devices for on-site, multiplexed detection of foodborne contaminants, thereby enhancing practicality and accessibility in real-world food monitoring.

#### 6.2.3. Modular Integration Strategies for Multipollutant Detection

In environmental monitoring, detection of a single pollutant is no longer sufficient to support comprehensive risk assessment. Microfluidic integrated systems [81], through multi-channel parallel designs, enable the simultaneous detection of multiple contaminants (Figure 4E). By incorporating precise fluidic control strategies, these platforms can reduce signal delay and extend the operational lifespan of sensing systems. Such configurations enhance analytical throughput while maintaining spatial separation between sensing modules, thereby minimizing cross-interference. In addition, the integration of allosteric transcription factors with optical fiber sensing technology has led to the development of portable water pollution analyzers, highlighting the advantages of optical carriers for in situ environmental detection. To detect heavy metals in the environment, researchers developed a portable, handheld, on-site detection platform that enables quantitative in situ analysis of Cd^2+^ and Hg^2+^ in water samples (Figure 4F) [91]. These approaches are not limited to a single class of analytes and can be adapted to diverse targets. Their modular design principles provide a scalable framework for constructing whole-cell biosensors that simultaneously detect multiple polycyclic aromatic hydrocarbons and their halogenated derivatives, thereby advancing toward integrated, field-deployable environmental surveillance platforms.

### 6.3. Biosafety, Regulatory, and Societal Considerations for Environmental Deployment

Engineered whole-cell biosensors based on genetically modified microorganisms (GMMs) have enabled a wide range of environmental applications, including pollutant degradation, agricultural enhancement, self-healing materials, and living therapeutic systems [92]. However, the potential environmental release of these engineered systems has raised increasing concerns regarding microbial persistence, horizontal gene transfer, and ecological impacts, highlighting the need for robust biosafety control strategies. From a design perspective, researchers have developed multiple genetic biocontainment approaches, including kill switches [93], auxotrophic dependencies [94], and inducible genetic circuits, to restrict the persistence and unintended dissemination of engineered microorganisms in natural environments. In addition, metabolic and functional engineering of microorganisms to enhance their ability to efficiently degrade environmental pollutants provides a scientific basis for safer environmental deployment of these systems. Nevertheless, the increasing complexity of synthetic genetic circuits and programmable microbial systems, while enhancing functional performance, also introduces greater biosafety uncertainty and consequently intensifies regulatory scrutiny.

This increasing regulatory scrutiny is further compounded by existing limitations in current governance frameworks for engineered microorganisms [95]. Existing regulatory frameworks often face classification ambiguity, as engineered microorganisms do not fit neatly into traditional categories such as pesticides, pharmaceuticals, or agricultural chemicals. Moreover, due to limited empirical evidence on long-term environmental persistence and ecosystem-level impacts, precautionary assumptions are frequently adopted in risk assessment, potentially delaying practical implementation. In addition, significant heterogeneity exists across national regulatory systems, and the number of approved large-scale environmental releases of genetically modified microorganisms remains limited, further complicating global deployment strategies.

To overcome these limitations, future regulatory and biosafety frameworks are expected to shift toward more adaptive and data-driven paradigms, enabling more accurate assessment of microbial survival, dispersal dynamics, and ecological impacts under realistic environmental conditions. Importantly, strengthening integration among scientists, policymakers, and the public will be essential to balancing technological innovation with ecological safety, thereby promoting transparent governance and the responsible development of engineered microbial technologies.

## 7. Summary and Perspectives

Overall, the development of whole-cell biosensors targeting PAHs and their halogenated derivatives has progressed from transcription-regulation-based constructs to modular optimization and, more recently, to exploratory translational applications. Advances in chassis cell selection, sensing element engineering, reporter system refinement, and the reinforcement of metabolic coupling strategies have collectively improved sensitivity, response specificity, and signal amplification efficiency, thereby gradually establishing a more systematic design framework. However, from a broader developmental perspective, a substantial gap remains between laboratory-level optimization and stable operation in complex environmental settings or large-scale deployment. Whole-cell biosensors inherently rely on living systems. While metabolic responsiveness and specificity confer high sensitivity, they also introduce signal variability and limited reproducibility. Moreover, the low solubility and strong hydrophobicity of PAHs and their halogenated derivatives make detection outcomes highly dependent on bioavailability rather than total environmental burden, potentially leading to structural bias in risk interpretation. A further critical limitation is the absence of unified performance evaluation standards and standardized testing frameworks. The lack of comparability across studies restricts effective pathway selection, benchmarking, and iterative optimization. Despite these challenges, the future development of whole-cell biosensors remains highly promising and dynamic. Continued integration of synthetic biology, systems engineering, and standardized validation strategies will be essential to unlocking their full potential for reliable, field-deployable environmental monitoring. Beyond technical performance, biosafety constraints, regulatory uncertainty, and societal acceptance remain key determinants for the successful environmental deployment of whole-cell biosensors.

Future development should not remain confined to incremental optimization at the component level but instead shift toward system-level engineering reconstruction and standardized integration. Enhancing overall robustness and predictability, establishing quantifiable and comparable performance benchmarks, and achieving deep coupling between design strategies and application scenarios are essential to advancing the field from proof-of-concept demonstrations to practical implementation. Whether the structural tension between biological complexity and engineering controllability can be effectively reconciled will ultimately determine the real value of whole-cell biosensors in field monitoring. With the continued convergence of synthetic biology, microsystems engineering, and intelligent computational platforms, whole-cell biosensors may evolve from static detection tools into distributed environmental sensing networks endowed with self-regulation, adaptive renewal, and cooperative perception capabilities. Such systems could enable high-throughput, real-time, and fine-grained risk identification in complex pollution contexts. This paradigm shift, from constructing individual biosensors to deploying intelligent sensing infrastructures, may ultimately define the strategic role of whole-cell biosensing technologies in future environmental monitoring and precision governance.

## Data Availability

No new data were created or analyzed in this study.
